# Adenomyosis and Endometrial Carcinoma—Prognostic Significance of Coexistence. A Retrospective Analysis

**DOI:** 10.1155/ogi/1883874

**Published:** 2026-09-17

**Authors:** Dina Gumin, Inshirah Sgayer, Alejandro Liboff, Tania Zlotkin, Ala Aiob, Susana Mustafa, Lior Lowenstein, Avishalom Sharon

**Affiliations:** ^1^ Department of Obstetrics and Gynecology of Raya Strauss, Galilee Medical Center, Nahariya, Israel, gmc.org.il; ^2^ Azrieli Faculty of Medicine, Bar-Ilan University, Safed, Israel, biu.ac.il; ^3^ Department of Pathology, Galilee Medical Center, Nahariya, Israel, gmc.org.il

**Keywords:** adenomyosis, endometrial carcinoma, molecular markers, prognosis

## Abstract

**Objectives:**

Although frequently identified in hysterectomy specimens of women with endometrial carcinoma, the impact of adenomyosis on the prognosis of endometrial carcinoma remains uncertain. Therefore, we aimed to evaluate the prognostic significance of coexistent adenomyosis and endometrial carcinoma, focusing on histopathological features, staging, and recurrence rates.

**Methods:**

We conducted a retrospective analysis of 142 women treated during 2013–2023 for endometrial carcinoma, categorized as Type 1 or Type 2. Clinical, histopathological, and recurrence data were compared between those with and without adenomyosis.

**Results:**

Adenomyosis was identified in 32/142 women (22.5%). Among 119 with Type 1 endometrial carcinoma, 26 (21.8%) had adenomyosis. FIGO stage I–II disease was present in all of these and in 76/93 (81.7%) of those without adenomyosis (*p* = 0.022). Cervical stroma was less frequently involved in the adenomyosis group (1/26; 3.8% vs 17/93; 18.3%, *p* = 0.05). Among the 23 women with Type 2 endometrial carcinoma, stage and recurrence rates did not differ significantly between those with and without adenomyosis. In histopathological assessment, malignant cells within adenomyotic foci were identified in six patients: five with Type 1 and one with Type 2 carcinoma. All of these involved deep diffuse adenomyosis. Strong PAX8 positivity was found in four women, and estrogen receptor positivity in five.

**Conclusion:**

We report an independent association of adenomyosis in Type 1 endometrial carcinoma with earlier‐stage disease and reduced cervical stroma involvement. These findings highlight a potential association between adenomyosis and favorable tumor characteristics, warranting further studies to clarify the prognostic significance.

## 1. Introduction

Endometrial carcinoma is the most common gynecologic malignancy in developed countries [1]. The prognosis is primarily determined by stage, histological subtype, and molecular characteristics [1]. Most of the patients present with early‐stage disease, particularly estrogen‐dependent endometrioid carcinomas, which generally have favorable outcomes. Key prognostic factors include lymphovascular space invasion and molecular markers like p53 mutations, both of which are associated with adverse outcomes [2]. Rising incidence and mortality rates of endometrial carcinoma in recent years are likely due to increases in risk factors [3]. Various guidelines incorporating histopathological and molecular features have been proposed to refine prognostic assessments and guide treatment strategies [2, 3]. Advances in oncogenomics, such as *The Cancer Genome Atlas* molecular subtyping, further emphasize the importance of tailored therapeutic approaches [4].

Adenomyosis, characterized by the presence of endometrial stroma and glands within the myometrium, is a common uterine condition. The two main forms, diffuse and focal, are further categorized as superficial or deep, according to the depth of myometrial invasion [5]. Adenomyosis affects 10%–80% of premenopausal women and is associated with symptoms like menorrhagia, abnormal uterine bleeding, dysmenorrhea, infertility, and spontaneous abortions [5]. Adenomyosis appears frequently in hysterectomy specimens of women with endometrial carcinoma. Prevalence has been reported in the range of 10%–34%, and particularly high in women over 55 years of age [6].

Despite the common coexistence of endometrial carcinoma and adenomyosis, data on their interrelations remain inconclusive and conflicting. Some have suggested that adenomyosis may be associated with better outcomes in early‐stage endometrial carcinoma, with a higher proportion at FIGO stage I. However, these patients often exhibit deeper myometrial invasion, possibly due to the increased surface area for tumor infiltration in the adenomyotic myometrium [7]. Various theories have been suggested regarding the prognostic impact of adenomyosis. Some have proposed that its antitumoral cytokine profile, thickened stromal tissue acting as a barrier, and early symptom‐driven detection may confer a better prognosis. Conversely, others have suggested that increased myometrial invasion, lymphovascular spread, malignant transformation, and reduced imaging accuracy may negatively affect outcomes [8].

Given the unclear relationship between adenomyosis and the prognosis of endometrial carcinoma, the high incidence of co‐existence, and the rising incidence and mortality of endometrial carcinoma, we aimed to assess the prognostic value of coexistent adenomyosis in patients with endometrial carcinoma. To this end, we compared, among patients with endometrial carcinoma, the histopathological features, staging, and recurrence rates between those with and without adenomyosis treated at our center during 2013–2023. We also tried to characterize the adenomyotic foci on purpose to find a relationship between different types of adenomyosis and the natural course of neoplasia. Additionally, we collected available data on immunohistochemical staining for cancers in order to find the predominance of any of them in cases of co‐existence with adenomyosis.

## 2. Materials and Methods

### 2.1. Study Design and Data Collection

This is a retrospective analysis of women treated in the Gynecology Department of the Galilee Medical Center, Nahariya, from January 2013 to November 2023. We examined all the records of women with a histological diagnosis of Type 1 or 2 endometrial carcinoma, carcinosarcoma, or sarcoma who underwent a total hysterectomy. The cohort was divided into two groups according to pathological findings: women with coexisting adenomyosis and uterine malignancy and women with uterine malignancies only.

The collected data included demographic information, the presence of bleeding at presentation, the type of biopsy, dates of biopsy and final surgery, complete medical history, pathological findings including cancer staging, immunohistochemical features, family history of malignancy, and co‐existing findings. Data on family history included ovarian, breast, uterine, colon, and other cancers. Co‐existing findings included adenomyosis, myomas, endometrial hyperplasia, and atrophy. Microscopic criteria were used for the pathological diagnosis of adenomyosis by our experts in the Department of Pathology [9]. We used the 2009 FIGO classification [10] and FIGO microscopic grading [2] for staging endometrial carcinoma. Additionally, we collected results of immunohistochemical staining performed as part of pathological evaluations, including MSH2, MSH6, MLH1, PMS2, PAX8, ER, PR, and P53. It is important to note that, due to the retrospective design of the study, the dominant data were collected from the period when the previous FIGO classification still was used, and molecular staining wasn’t a routine part of every histological evaluation. In this regard, we expected inconsistencies in the data.

The primary outcome of the research was to find if adenomyosis can be an underlying disease for Type 1 and Type 2 endometrioid carcinoma and to evaluate its relationship to presenting symptoms, histology, grade, and the prognosis. As secondary outcomes, we wanted to analyze the location of adenomyotic cells and the presence of malignant cells in the adenomyosis in relation to the type of carcinoma. Also, the relation to demographic and anamnestic characteristics and the presence of different molecular mutations in immunohistochemical staining were analyzed.

### 2.2. Statistical Analysis

Univariate analysis was used to compare between groups. Statistical analysis involved presenting quantitative variables as means ± standard deviations or as medians and interquartile ranges. Qualitative variables were presented as frequencies and percentages. Quantitative data were compared using the independent samples *t*‐test or the Wilcoxon rank‐sum test. Qualitative variables were compared using the chi‐square test or Fisher’s exact test, as appropriate. A *p*‐value of less than 0.05 was considered statistically significant. Statistical analysis was performed using IBM SPSS Statistics, version 27.

## 3. Results

### 3.1. The Study Population

During the study period, 172 women presented with uterine corpus malignancies of different types. All the patients underwent hysterectomy after getting biopsy results suspicious for any malignant neoplasia of the uterus. All the cases were included regardless of the managing surgeon and the presence of malignancy in the final pathological sampling. Patients who didn’t perform the hysterectomy or underwent it in other medical centers weren’t included in the analysis. Moreover, women who underwent hysterectomy for other types of gynecological malignancy (ex., ovarian cancer or cervical cancer) were not included in this research. All the patients were at least 18 years old at the time of surgery.

**FIGURE 1:**
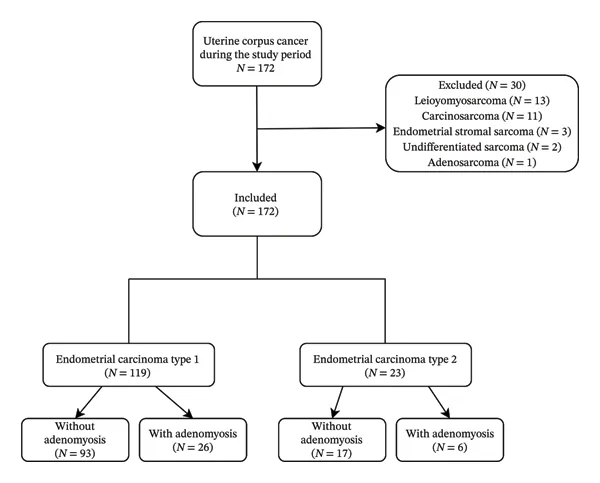
A flow diagram of the study selection process.

Of these, 142 were classified as having endometrial carcinoma, and 30 with other types of malignancies, including sarcomas and carcinosarcomas. These 30 were not included in the statistical analysis due to their small number and the presence of adenomyosis in only two of them. Figure 1 presents a flow diagram of the study selection process.

Among the 142 women with endometrial carcinoma, 32 (22.5%) had adenomyosis. Of the 119 endometrioid (Type 1) carcinomas, 26 (21.8%) were associated with adenomyosis. Among the 23 nonendometrioid (Type 2) carcinomas, 6 (26.1%) were associated with adenomyosis. The analysis was conducted separately for Type 1 and Type 2 endometrial carcinomas due to their distinct nature, etiology, therapeutic approaches, and prognoses.

### 3.2. Type 1 Endometrial Carcinoma

Patients with and without adenomyosis were similar in age, smoking rate, number of births, age of menopause, and time from menopause to diagnosis of carcinoma (Table 1). The groups did not differ significantly in factors relating to the patients’ medical histories. The median body mass index was higher among the patients with adenomyosis than without (40.0 vs. 31.6, *p* = 0.026). This finding can suggest that obesity is an additional risk factor for the development of adenomyosis. On the other hand, the observed difference indicates that the groups were not fully comparable at baseline, which may introduce confounding and complicate the statistical analysis and interruption of the results.

**TABLE 1:** Demographic and anamnestic features of women with endometrial carcinoma Type 1 and Type 2, with and without adenomyosis.

Variable	Type 1 (*N* = 119)		*p* Value	Type 2 (*N* = 23)		*p* value
	Without adenomyosis (*N* = 93)	With adenomyosis (*N* = 26)		Without adenomyosis (*N* = 17)	With adenomyosis (*N* = 6)	
Demographic and anamnestic features						
Age (years), mean (±SD)	64.3 (±11.5)	65.15 (±11.2)	0.747	70.0 (±10.0)	69.7 (±10.3)	0.85
BMI (kg/m^2^), median (IQR)	31.6 (26.0–36.6)	40.0 (32.4–42.9)	0.026	35.2 (28.5–38.1)	34.2 (29.4–39.0)	
Smoking	8 (13.6%)	13 (18.8%)	0.692	0 (0%)	0 (0%)	
Bleeding						
None	13 (14.0%)	2 (7.7%)		4 (23.5%)	1 (16.7%)	
Menorrhagia	4 (4.3%)	2 (7.7%)		0 (0%)	0 (0%)	
Menometrorrhagia	5 (5.4%)	0 (0%)		1 (5.9%)	0 (0%)	
Postmenopausal bleeding	68 (73.1%)	22 (84.6%)	0.599	12 (70.6%)	5 (83.3%)	1.000
Irregular bleeding	3 (3.2%)	0 (0%)		0 (0%)	0 (0%)	
Underlying diseases	80 (86.0%)	20 (76.9%)	0.36	17 (100.0%)	4 (66.7%)	0.059
Chronic hypertension	61 (65.8%)	16 (61.5%)	0.817	15 (88.2%)	1 (16.7%)	0.003
Diabetes mellitus	39 (41.9%)	11 (42.3%)	1	7 (41.2%)	2 (33.3%)	1
Hyperlipidemia	39 (41.9%)	6 (23.1%)	0.109	8 (47.1%)	2 (33.3%)	0.66
Hypothyroidism	17 (18.3%)	2 (7.7%)	0.24	2 (11.8%)	0 (0%)	1
Other malignancy	11 (13.4%)	1 (4.3%)	0.457	1 (6.7%)	0 (0%)	1
Age of menopause (years), median (IQR)	50 (44.5–52.5)	51 (47.8–53.0)	0.557	50 (45.0–53.5)	50 (47.5–52.0)	0.909
Time from menopause to carcinoma (years), median (IQR)	14 (7.0–23.5)	12.5 (4.3–27.3)	0.944	21.5 (10.8–29.3)	15 (9.5–11.2)	0.625
Median (IQR) number of births	2 (0–3)	2 (1–3)	0.708	2 (1–7)	3 (2–5)	0.896
Family history of malignancy						
None	53 (71.6%)	10 (52.6%)	0.075	12 (70.6%)	4 (80.0%)	0.388
Gynecology‐related	9 (12.2%)	5 (26.3%)		4 (23.5%)	0 (0%)	
Not gynecology‐related	9 (12.2%)	1 (5.3%)		1 (5.9%)	1 (20.0%)	
Both	3 (4.1%)	3 (15.8%)		0 (0%)	0 (0%)	

**FIGURE 2:**
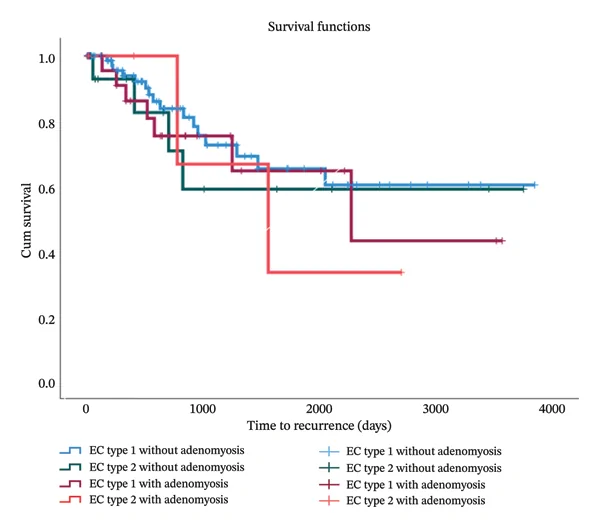
Kaplan–Meier estimator for recurrence of endometrial carcinoma Type 1 and Type 2 in women with and without adenomyosis. EC = endometrial carcinoma.

Table 2 presents the pathological features of the 119 patients with Type 1 endometrial carcinoma, comparing the 26 with and the 93 without adenomyosis. Limited disease (Stages 1 and 2) was more common among those with than without adenomyosis (100% vs. 81.7%, *p* = 0.022). Extensive disease (Stages 3 and 4) was found only in patients without adenomyosis. Cervical stroma was less frequently involved in those with than without adenomyosis (3.8% vs. 18.3%, *p* = 0.05). The groups did not differ in other pathological features, such as lymphovascular space invasion, metastasis, or cytology positivity. Similarly, clinical features, including tumor grade, recurrence rates, and adjuvant treatments such as brachytherapy, external radiation, chemotherapy, and combined chemoradiation, did not differ significantly between the groups. Among the patients with type 1 endometrial cancer, the types and frequencies of adjuvant treatment and recurrence did not differ between those with and without adenomyosis (Figure 2).

**TABLE 2:** Pathological findings of women with endometrial carcinoma Type 1 and Type 2, with and without adenomyosis.

Variable	Type 1 (*N* = 119)		*p* Value	Type 2 (*N* = 23)		*p* value
	Without adenomyosis (*N* = 93)	With adenomyosis (*N* = 26)		Without adenomyosis (*N* = 17)	With adenomyosis (*N* = 6)	
Pathological features						
Depth of invasion			0.658			1
Less than 50%	47 (50.5%)	15 (57.7%)		7 (41.2%)	3 (50.0%)	
50% or more	46 (49.5%)	11 (42.3%)		10 (58.8%)	3 (50.0%)	
Invasion of other pelvic structures						
Cervical stroma	17 (18.3%)	1 (3.8%)	0.056	6 (35.3%)	2 (33.3%)	1
Ovaries	6 (6.5%)	0 (0%)	0.337	3 (17.6%)	1 (16.7%)	1
Tubes	5 (5.4%)	0 (0%)	0.584	1 (5.9%)	2 (33.3%)	0.155
Parametrium	11 (11.8%)	1 (3.8%)	0.46	5 (29.4%)	3 (50.0%)	0.6221
Lymph nodes						
None	66 (86.8%)	20 (100%)	0.364	12 (75.0%)	3 (50.0%)	0.334
PLN	9 (11.8%)	0 (0%)		4 (25.0%)	3 (50.0%)	
PALN	1 (1.3%)	0 (0%)		0 (0%)	0 (0%)	
Metastasis	6 (7.3%)	1 (5.6%)	1	2 (12.5%)	0 (0%)	1
LVSI positive	12 (16.4%)	4 (20.0%)	0.742	6 (40.0%)	2 (33.3%)	1
Positive cytology	11 (15.7%)	1 (5.3%)	0.449	1 (7.1%)	1 (20.0%)	0.468
Clinical features						
Grade			0.868			
1	38 (40.9%)	11 (42.3%)				
2	32 (34.4%)	10 (38.5%)				
3	23 (24.7%)	5 (19.2%)				
Stage						
Limited disease (Stage 1 + 2)	76 (81.7%)	26 (100%)	0.022	10 (58.8%)	2 (33.3%)	0.371
Extensive disease (Stage 3 + 4)	17 (18.3%)	0 (0%)		7 (41.2%)	4 (66.7%)	
Adjuvant treatment						
Brachytherapy	30 (36.6%)	9 (39.1%)	1	8 (53.3%)	4 (100%)	not comparable
External radiation	4 (4.9%)	1 (4.3%)	1	1 (6.7%)	0 (0%)	
Chemotherapy	7 (8.5%)	2 (8.7%)	1	5 (33.3%)	0 (0%)	
Chemotherapy + radiation	13 (15.9%)	1 (4.3%)	0.295	4 (26.7%)	4 (100%)	
Recurrence	16 (18.8%)	7 (28.0%)	0.401	4 (26.7%)	2 (40.0%)	0.613

*Note:* LVSI lymphovascular space invasion.

Abbreviations: PALN, para‐aortic lymph nodes; PLN, pelvic lymph nodes.

### 3.3. Type 2 Endometrial Carcinoma

Patients with and without adenomyosis were similar in the parameters examined, except for the rate of chronic hypertension (Table 2), which was more prevalent among those without adenomyosis (88.2% vs. 16.7%, *p* = 0.003). Statistically significant differences were not found between the groups in the depth of myometrial invasion, the involvement of other pelvic structures, or cytology positivity (Table 2). Similarly, differences were not observed in tumor grade, disease stage (limited vs. extensive), recurrence rates, or the use of adjuvant treatments. Nonetheless, the small number of patients in this group limits the statistical analysis and precludes any definitive conclusions.

### 3.4. Histological Assessment

We reviewed all the histological samples to characterize adenomyotic foci and their association with tumor cells. A senior pathologist conducted the review following internationally accepted microscopic criteria. Specifically, we focused on two pathological details: the extent of adenomyosis and the presence of malignant cells within adenomyotic foci. Based on these findings, adenomyotic foci were categorized as superficial (junctional zone adenomyosis) or deep and further classified as focal or diffuse.

Of the 32 women diagnosed with adenomyosis, secondary review did not reveal adenomyotic foci in three. These three were not excluded from the statistical analysis, as this discrepancy may have been due to technical issues, such as missing preparations in the archive or the transfer of samples to other laboratories for additional studies. The results are summarized in Table 3. Deep adenomyosis was observed in 23 of 29 (79.3%), and diffuse adenomyosis in 19 of 29 (65.5%). Malignant cells within adenomyotic foci were identified in 6 of 23 (26.1%) of those with deep diffuse adenomyosis. These comprised five with Type 1 carcinoma and one with type 2 carcinoma. Among those with Type 1 carcinoma, three were FIGO grade 1, and two were FIGO Grade 2. Table 4 presents the FIGO grades and stages for these six patients. Four demonstrated deep myometrial invasion (involving more than half the myometrial thickness), and one exhibited local spread with pelvic lymph node involvement.

**TABLE 3:** Histological features of adenomyosis in women with endometrial carcinoma Type 1 and Type 2.

Case number	Type of carcinoma	Depth: superficial‐0 deep‐1	Degree of extension: focal‐0 widespread‐1	Presence of malignant cells in focus: no – 0 yes – 1
1	Type 1	1	1	0
2	Type 1		No adenomyosis	
3	Type 1	0	0	0
4	Type 1	1	1	1
5	Type 2	0	0	0
6	Type 1	0	0	0
7	Type 2	1	1	0
8	Type 2	1	0	0
9	Type 1	1	1	1
10	Type 1	1	1	1
11	Type 1	0	0	0
12	Type 1	1	1	0
13	Type 1		No adenomyosis	
14	Type 1	1	1	0
15	Type 1	1	1	0
16	Type 2	1	1	1
17	Type 1	1	1	0
18	Type 1	1	0	0
19	Type 1		No adenomyosis	
20	Type 1	1	1	0
21	Type 1	0	0	0
22	Type 1	0	0	0
23	Type 1	1	0	0
24	Type 1	1	1	0
25	Type 1	1	1	1
26	Type 1	1	1	1
27	Type 1	1	1	0
28	Type 2	1	1	0
29	Type 1	1	1	0
30	Type 1	1	1	0
31	Type 1	1	0	0
32	Type 2	1	1	0

**TABLE 4:** FIGO grades and stages of the malignancies that arose within adenomyotic foci.

Case	Age	Type	Grade	Stage
1	58	1	1	1b
2	61	1	2	1b
3	68	1	1	1b
4	77	2	3	3c1
5	65	1	2	1a
6	68	1	2	1a

### 3.5. Immunohistochemical Staining

The additional data about molecular staining were collected for all the patients with and without coexistent adenomyosis, including MSH2, MSH6, MLH1, PMS2, PAX8, ER, PR, and P53. The results were available only for 32 of 142 cases, and for most of them were partial. Due to limited data, it was impossible to perform statistical analyses for the results, but some trends were noticed in the group of patients with ECC and adenomyosis.

In four of the six for whom immunohistochemical staining was performed, the tumor exhibited strong positivity for PAX8. In five, including the patient with type 2 malignancy, strong estrogen receptor positivity was observed. The retrospective design precluded performing immunohistochemical staining on all the samples, and the available data were insufficient for statistical analysis.

## 4. Discussion

Among 142 women with endometrial carcinoma, 32 (22.5%) had adenomyosis; that is, 21.8% for Type 1 endometrial carcinoma and 26.1% for Type 2. In Type 1 carcinomas, adenomyosis was associated with earlier stage disease and less cervical stroma involvement, while statistically significant differences were not observed in recurrence rates or treatment approaches. At the same time, we didn’t find this association for the Type 2 carcinomas. Deep diffuse adenomyosis was found in about 2/3 of all the patients with co‐existence of adenomyosis and endometrial carcinoma (79.3% and 65.5%, respectively). Malignant cells within the endometrial foci were detected in 5 patients with the first type of carcinoma, and all the women had FIGO Stage 1 or 2 disease.

Our report of adenomyosis in 22.5% of patients with endometrial carcinoma is consistent with the 22.6% prevalence reported in a systematic review by Raffone et al. [11]. Similarly, prevalences in the range of 21%–36% were reported for other gynecological conditions, such as uterine fibroids, endometrial hyperplasia, and abnormal uterine bleeding [12, 13]. These similarities suggest that the coexistence of adenomyosis and endometrial carcinoma is likely driven by shared demographic and hormonal factors, such as prolonged estrogen exposure, rather than by a unique pathological link [14]. Adenomyosis is commonly diagnosed in peri‐ and postmenopausal women and is associated with the same demographic factors that are most affected by endometrial carcinoma, thus further supporting the overlap between the conditions. While the inflammatory and hormonal environment associated with adenomyosis may influence endometrial pathology, evidence suggests that the coexistence reflects common risk factors rather than a direct causal relation [14].

Among our patients with endometrial carcinoma, those with coexistent adenomyosis had a higher proportion diagnosed at earlier FIGO stages (I and II) and a lower proportion with cervical stromal involvement. These findings align with reported associations of adenomyosis with favorable tumor characteristics [14, 15]. For example, An et al. [15] reported that adenomyosis in women with endometrial carcinoma was associated with reduced deep myometrial invasion (odds ratio (OR) 0.45; 95% confidence interval (CI) 0.33–0.60; *p* < 0.00001). Additional associations of that study were lower lymphovascular space involvement (OR 0.44; 95% CI 0.29–0.68; *p* = 0.0002), and FIGO stage I–II compared to III–IV tumors (OR 1.85; 95% CI 1.49–2.30; *p* < 0.00001). Similarly, Raimondo et al. [16] found that coexistent adenomyosis was linked to early‐stage disease and improved overall survival. This is likely due to the association of adenomyosis with less invasive tumor behavior. Several theories have been proposed to explain these findings. Some have suggested that adenomyosis creates a mechanical barrier to tumor invasion, as the ectopic endometrium within the myometrium may limit tumor spread. Additionally, the altered cytokine profile in adenomyosis, characterized by increased antitumoral cytokines such as interferon‐γ and tumor necrosis factor‐α and by decreased oncogenic cytokines such as interleukin‐8, may inhibit tumor progression [14, 16]. It has also been speculated that the thickened endometrial stroma resulting from repeated inflammation in adenomyosis may further block tumor invasion [14]. Conversely, others argued that the large interface between adenomyosis and the myometrium could facilitate deeper tumor infiltration or lymphovascular spread, potentially worsening outcomes. Moreover, reduced imaging accuracy in assessing the depth of invasion in adenomyotic uteri might lead to under‐staging, thereby affecting treatment decisions [14, 17]. These contrasting theories highlight the complex interplay between adenomyosis and tumor behavior in endometrial carcinoma, warranting further research.

Distinct differences have been identified between endometrial cancer that arises in adenomyosis and endometrial cancer that coexists with adenomyosis. Endometrial cancer that arises in adenomyosis is more frequently associated with older age, non‐endometrioid histology, deep myometrial invasion, and advanced‐stage disease. Despite similar tumor grade and nodal metastasis risk, this cancer is linked to worse 5‐year disease‐free survival, 72.2%, compared to 85.5% for cancer coexisting with adenomyosis [18, 19]. The aggressive behavior of endometrial cancer arising in adenomyosis may stem from its origin in the myometrium, which facilitates lymphovascular spread [18]. In contrast, cancer coexisting with adenomyosis may benefit from the mechanical barrier provided by adenomyosis and its antitumoral cytokine environment, thus contributing to improved prognosis [14]. In about one‐quarter of our patients with deep diffuse adenomyosis, malignant cells were identified within adenomyotic foci. These were predominantly Type 1 carcinomas of low‐to‐moderate grade (FIGO Grades 1 and 2). While these findings suggest an association with lower grade carcinomas, deep myometrial invasion was observed in four patients and pelvic lymph node involvement in one. Although our study comprised a relatively small number of patients, the findings corroborate the aforementioned studies. Together, they support the notion that cancers arising within adenomyotic foci retain the potential for aggressive behavior despite the localized microenvironment of adenomyosis.

Over the past decade, molecular classification has revolutionized the diagnostic and therapeutic approach to endometrial carcinoma, as markers such as POLE Mut, MMRd, NSMP, KRAS, and p53abn have become integral to FIGO staging 2023 and treatment strategies. Emerging evidence has linked PAX8 overexpression with p53 expression and high‐grade endometrial carcinomas and thus to a worse prognosis and more aggressive tumor behavior [20–22]. While molecular testing was not routinely implemented during the period of our study, we collected available pathological data and noted a potential association between adenomyosis and PAX8‐positive tumors. Although the data were insufficient for definitive conclusions, this observed trend highlights the need for further research into the role of PAX8 in the prognosis of endometrial carcinoma.

### 4.1. Strengths and Limitations

The long study period (2013–2023) is a strength of the study. Moreover, the single‐center design ensured consistent treatment protocols and follow‐up practices. The comprehensive analysis and comparison contribute insights into the clinical and pathological characteristics of endometrial carcinoma and its association with adenomyosis through detailed examination of staging, recurrence, and pathological features. However, the study has limitations. Its retrospective design relied on existing pathological reports, which may have inconsistencies or missing data. The relatively small sample size, particularly of carcinomas within adenomyotic foci, and no multivariate analysis limit the generalizability of the findings. Additionally, hysterectomy is not the first‐line treatment for some patients with advanced disease (Stages 3 and 4). Some of them will be referred to neo‐adjuvant treatment prior to the surgery, which can affect the histological findings and the results of the study; some won’t undergo hysterectomy at all. Furthermore, molecular data were incomplete, as testing was not routinely performed during the earlier years of the study. This restricts insights into molecular pathways.

## 5. Conclusion

To conclude, this study adds to the existing literature in its exploration of the relation between adenomyosis and endometrial carcinoma. Although only univariate analyses were performed, the results testify in favor of the potential association with earlier stages of the disease in Type 1 endometrial carcinoma. However, further research is required to determine whether adenomyosis acts as an independent prognostic factor and to better understand its influence on tumor behavior, staging, and outcomes, with a focus on the prognostic role of adenomyotic features and the relationship with variable molecular mutations.

## Author Contributions

Dina Gumin—writing, data curation, and formal analysis; Inshirah Sgayer—writing, and review and editing; Alejandro Liboff and Tania Zlotkin—validation and data curation; Ala Aiob—review and editing, and formal analysis; Susana Mustafa—data curation and formal analysis; Lior Lowenstein and Avishalom Sharon—conceptualization, and review and editing.

## Funding

No funding was received for this research.

## Ethics Statement

This study was approved by the Ethics Committee of Galilee Medical Center, Nahariya, Israel (IRB approval number: The protocol of the study was approved by the local Institutional Review Board (Helsinki Committee) of the Galilee Medical Center, XX (number of approval NHR‐23‐0050)). Due to the retrospective nature of the study and the use of anonymized patient data, the institutional review board waived the requirement for informed consent.

## Consent

Ethics approval was obtained, and the requirement for individual consent was waived by the institutional review board, as detailed above.

## Conflicts of Interest

The authors declare no conflicts of interest.

## Data Availability

The datasets used and analyzed during the current study are available from the corresponding author on reasonable request.
